# Dissecting the Vesicular Trafficking Function of IFT Subunits

**DOI:** 10.3389/fcell.2019.00352

**Published:** 2020-01-15

**Authors:** Huihui Yang, Kaiyao Huang

**Affiliations:** ^1^Key Laboratory of Algal Biology, Institute of Hydrobiology, Chinese Academy of Sciences, Wuhan, China; ^2^Institute of Hydrobiology, University of Chinese Academy of Sciences, Beijing, China

**Keywords:** cilium, flagellum, IFT, vesicular trafficking, ciliopathy

## Abstract

Intraflagellar transport (IFT) was initially identified as a transport machine with multiple protein subunits, and it is essential for the assembly, disassembly, and maintenance of cilium/flagellum, which serves as the nexus of extracellular-to-intracellular signal integration. To date, in addition to its well-established and indispensable roles in ciliated cells, most IFT subunits have presented more general functions of vesicular trafficking in the non-ciliated cells. Thus, this review aims to summarize the recent progress on the vesicular trafficking functions of the IFT subunits and to highlight the issues that may arise in future research.

## Introduction

The formation and function of the cell depend on the appearance of phospholipid membranes. During a crucial stage of prokaryote-to-eukaryote transition, various subcellular membrane-bound compartments appeared through folding, bending, and fusing phospholipid membranes, thus contributing to the organization of the endomembrane system (Dacks and Field, 2007). This process directly expands the total area of the membrane, thereby providing a scaffold for the attachment of proteins and lipids. It also divides the interior of the cell into several functional regions, which ensures a unique microenvironment suitable for specific biochemical reactions.

Nevertheless, compared with free diffusion in the prokaryotic cytoplasm, transport of soluble molecules and biomacromolecules is hampered by the hydrophobic structure of phospholipids in the endomembrane system. As a coevolutionary system, the dynamic and targeted vesicular transport has been adopted by eukaryotes. The endomembrane system comprises the nuclear envelope, rough and smooth endoplasmic reticulum (ER), Golgi apparatus, endosomes, and vesicles that bud off from these membrane structures. Golgi functions as the center of the endomembrane system, thus selectively accepting protein and lipid cargoes that are transported from the ER at the *cis-*Golgi, processing them at the medial-Golgi, and incorporating them into specific vesicles at the *trans-*Golgi site and *trans-*Golgi networks (TGNs) (Rothman, 1981). The underlying molecular mechanism of vesicular transport in the endomembrane system, such as vesicle budding, trafficking, tethering, and fusion, has been well studied and summarized (Cai et al., 2007; Hutagalung and Novick, 2011).

The cilium/flagellum, a membrane-bound organelle protruding from the cell surface and sharing several vesicular trafficking components with the endomembrane system, has gained immense attention due to its sensory, motile, and secretory roles in development, homeostasis, and ciliopathies (Bisgrove and Yost, 2006; Fliegauf et al., 2007; Long et al., 2016; Sanchez and Dynlacht, 2016; Reiter and Leroux, 2017). Similar to the other membrane-bound organelles, the cilium lacks protein synthesis machinery (Johnson and Rosenbaum, 1992). Therefore, a matched transport system is required to maintain the structure and function of the cilium. Until 1993, this transport system was observed by video-enhanced differential interference-contrast (DIC) microscopy and was termed intraflagellar transport (IFT), which can be imaged as bidirectional trains between the ciliary skeleton and membrane (Kozminski et al., 1993).

Furthermore, numerous studies helped in understanding the composition, assembly, and functions of the IFT system (Cole, 2003; Taschner et al., 2012; Lechtreck, 2015; Prevo et al., 2017). Evolutionarily, various components of a novel system are presumably the result of gene duplications, thereby producing proteins that have similar functions but differ in their subcellular locations (Dacks and Field, 2007). Phylogenetic evidence revealed that IFT complexes originated from the vesicle coats, which are the essential components of vesicular transport in the endomembrane system (Jekely and Arendt, 2006; van Dam et al., 2013). Based on this evidence, it is speculated that IFT appeared earlier and, subsequently, was co-opted by an ancestral eukaryote to establish the cilium. Recently, increasing evidence has revealed that several IFT subunits indeed have extraciliary sites, suggesting that they presumably played primitive vesicle-associated roles (Finetti et al., 2009, 2014; Finetti and Baldari, 2013; Noda et al., 2016). Herein, we mainly review the vesicular trafficking roles of IFT subunits in the ciliated and non-ciliated cells.

## The Vesicular Trafficking Function of IFT Subunits in Ciliated Cells

### Ciliogenesis

Cilia are highly conserved organelles and widely distributed from the unicellular green alga *Chlamydomonas* to most human cells. The assembly of cilia is believed to begin when the cells exit the mitotic cycle and can be roughly divided into the following phases. First, small cytoplasmic vesicles originating from the Golgi or recycling endosomes accumulate at the distal appendages (DAPs), a unique structure of the mother centriole. The centrosome comprises two mutually perpendicular centrioles (mother and daughter) that differ in their ultrastructure. The mother centriole can acquire the appendages at the distal end (a process known as centriole maturation), while the daughter cannot. Only the mature centriole can support ciliogenesis (Vorobjev and Chentsov Yu, 1982). Second, these vesicles fuse and produce a membranous cap on the distal end of the mother centriole. Third, this mother centriole, now called basal body, functions as a template to initiate the growth of the microtubule doublets/axoneme, the core of cilium. The extension of the mother centriole will be sheathed by the top membranous cap. Fourth, this nascent cilium docks to a specific patch of the plasma membrane through the DAPs structure, accompanying the fusion of the ciliary membrane cap with the plasma membrane. Subsequent vesicles are continually transported into the periciliary membrane compartment (PCMC), a transition membrane zone between the plasma and ciliary membranes (in some cell types, they are in pocket shape), and contribute in enlarging the ciliary membrane (Kaplan et al., 2012; Benmerah, 2013; Lu et al., 2015; Sanchez and Dynlacht, 2016).

Similar to other membrane-bound organelles, vesicular trafficking is indispensable for the cilia assembly; however, the cilium is not entirely membrane-surrounded. Blade-like structures derived from DAPs and termed transition fibers stretch across the membrane-unbound zone, tether the mother centriole to the periciliary membrane, and demarcate the entrance to the cilium. The transition zone (TZ) starts from above these transition fibers. Y-shaped structures characterize this TZ, connecting the ciliary membrane and microtubule doublet and acting as a filter or a gate presumably owing to the size exclusion in an already narrow cilium with ∼200 nm diameter (Craige et al., 2010; Kee et al., 2012). Although the ciliary membrane is continuous with the plasma membrane, the existence of transition fibers and TZ ensures that the cilium contains its unique membrane receptors and lipids for sensing and transmitting extracellular signals (Pazour et al., 2005; Goetz and Anderson, 2010; Hilgendorf et al., 2016).

### Intraflagellar Transport

The active transport system in the cilium, IFT, was initially discovered in *Chlamydomonas* (Kozminski et al., 1993). Furthermore, researches on other organisms, such as *Caenorhabditis elegans*, *Trypanosoma brucei*, *Tetrahymena*, sea urchin, zebrafish, mice, and human cell lines, have greatly enhanced the knowledge of IFT. In electron micrographs, IFT appears as a granule-like tightly apposed structure between the microtubule doublets and ciliary membrane, which comprises motors (kinesin II and cytoplasmic dynein 2) and IFT complexes serving as adaptors or bridges between the cargoes and motors (Kozminski et al., 1993, 1995).

**FIGURE 1:**
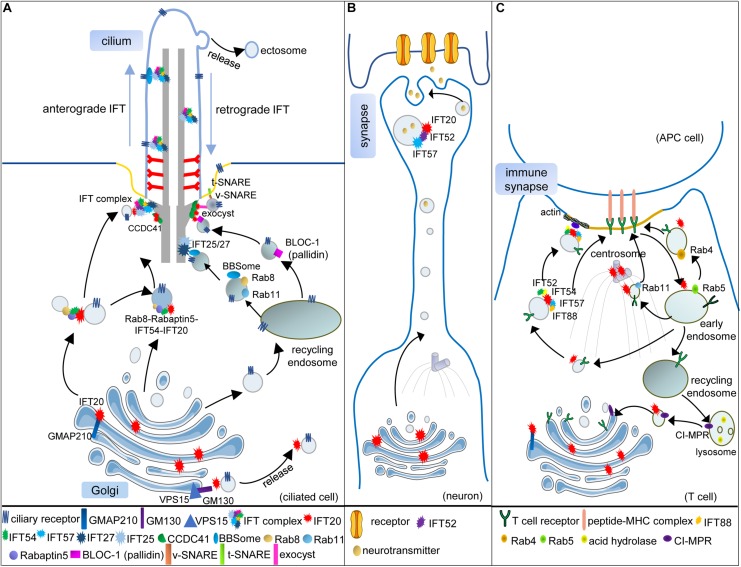
Vesicular trafficking function of IFT subunits in ciliated and non-ciliated cells. **(A)** In the ciliated cell, IFT20 localizes at both the cilium and the Golgi and transports the ciliary receptors from the Golgi to the cilium. Golgi resident protein GMAP210 recruits IFT20 at the Golgi. Another Golgi resident protein GM130, along with VPS15, regulates the release of IFT20-associated vesicles from the Golgi. IFT54 interacts with IFT20 and Rabaptin5, an effector of Rab8, to mediate the interaction of Rab8-Rabaptin5-containing and IFT20-containing vesicles when these vesicles are directed to the base of the cilium. At the basal body, these vesicles can be assembled with the other IFT subunits, and anterograde IFT trains transport ciliary receptors into the cilium. Some ciliary receptors could also be transported by recycling endosomes with the help of Rab8, Rab11, and BBSome. When the vesicles carrying Rab8, Rab11, and the BBSome reach the basal body, IFT25/27 interact with the BBSome to facilitate ciliary receptors incorporating into IFT trains. The BLOC-1 complex is also associated with the ciliary receptors in recycling endosome-derived vesicles. Moreover, IFT20 is partially responsible for the basal body localization of pallidin, one subunit of the BLOC-1 complex. Two components of the exocyst complex (Exo70 and Sec8) also interact with IFT20 at the basal body, which might facilitate the interaction of v-SNARE and t-SNARE. One component of DAPs, CCDC41, can recruit IFT20 to the basal body where several IFT subunits associated with vesicular trafficking are assembled into the whole IFT complex. **(B)** Vesicular trafficking functions of IFT20, IFT52, and IFT57 in the neuron. During the maturation of neurons, the microtubule organizing center (MTOC; centrosome) and Golgi are translocated toward the neurite. IFT20 localizes at the Golgi and transports synaptic vesicles along with IFT52 and IFT57 in polarized axons. **(C)** Vesicular trafficking functions of IFT subunits in T cells. When the T cell is activated, and the immune synapse (IS) begins to assemble, the (MTOC; centrosome) is translocated beneath the membrane domain of the IS. The Golgi and other vesicular compartments also relocate toward the IS. The T-cell receptors (TCRs) need to be transported to the IS for the activation of T cells. IFT20, localizing at the MTOC, Golgi, and endosomes, is required for polarized TCR recycling to the IS with the help of IFT52, IFT54, IFT57, and IFT88. Transmit of internalized TCRs from early endosomes (Rab5) to recycling endosomes (Rab4) or pericentrosomal recycling endosomes (Rab11) also needs IFT20. In addition, IFT20 also regulates the retrograde vesicular trafficking of the cation-independent mannose-6-phosphate receptors (CI-MPR) from lysosomes to the *trans-*Golgi network, thus controlling lysosome biogenesis in both ciliated and non-ciliated cells [only presented in **(C)**].

Intraflagellar transport complexes could be further fractionated into IFT-A and IFT-B subcomplexes. The IFT-A complex, comprising six subunits (IFT144/140/139/122/121, and 43), mediates the retrograde transport from the cilium tip to the basal body. The IFT-B complex, subdivided into a 10-subunit core IFT-B1 complex (IFT-88/81/74/70/56/52/46/27/25, and 22) and a 6-subunit peripheral IFT-B2 complex (IFT-172/80/57/54/38, and 20), is responsible for the anterograde transport from the basal body to the cilium tip (Figure 1A; Cole, 2003; Scholey, 2003).

To date, none of the IFT proteins possess transmembrane domains, lipid modifications, or lipid-binding domains, and vesicular trafficking is widely believed to be absent inside the cilia (Taschner et al., 2012); however, IFT subunits surprisingly share their domain organization with classical vesicular coat proteins (COPs), which identify various vesicular trafficking pathways in the endomembrane systems (Jekely and Arendt, 2006; van Dam et al., 2013). Structurally, the subunits of IFT complex are rich in the WD domain, tetratricopeptide (TPR) repeats, and coiled-coil (CC) domain, thus displaying innate potential for protein–protein interactions (Taschner et al., 2012; Lechtreck, 2015). Moreover, IFT-coated vesicles carrying axonemal proteins were observed at the ciliary base (Wood and Rosenbaum, 2014). In *C. elegans*, the movement of membrane channels showed a comparable rate to IFT in cilia (Qin et al., 2005). A recent study reported that IFT172 is a membrane-interacting protein with an ability to remodel large membranes into small vesicles by using the giant unilamellar vesicles *in vitro* (Wang et al., 2018). In addition to IFT172, other IFT subunits also possess a membrane-associated function during ciliogenesis. In the present review, we have discussed these individual IFT subunits in detail.

### IFT20 and Vesicular Trafficking

IFT20 is the smallest IFT subunit with two predicted coiled-coil domains at the C terminal. At moderate salt concentrations, IFT20, as well as another five subunits, dissociate from the stably associated core subcomplex (IFT-B1), and they are grouped into a peripheral subcomplex (IFT-B2) (Taschner et al., 2016); however, a peripheral position in the IFT-B complex does not indicate that IFT20 is dispensable for ciliogenesis. In contrast, IFT20 is crucial for cilium formation and might be a well-studied example to prove that IFT subunits participate in vesicular trafficking. IFT20 has a unique Golgi localization, in addition to the basal body and cilium where the other IFT subunits localize (Figure 1A). A piece of direct evidence suggests that the highly dynamic vesicles comprising IFT20-GFP move between the Golgi and the basal body (Follit et al., 2006).

#### IFT20 and the Golgi

The Golgi apparatus is a stack of distinct cisternae arranged from *cis* to *trans* positions. In several cell types, the colocalization pattern between IFT20 and different Golgi markers suggested that IFT20 is associated with both the *cis* and medial cisternae, but not extensively with the TGN (Follit et al., 2006). IFT20 is recruited to the Golgi by GMAP210, a member of golgins (Figure 1A; Follit et al., 2008). Golgins are predominantly coiled-coil proteins and typically anchored to the cytosolic face of the Golgi membrane to capture or tether the transport vesicles (Munro, 2011); however, unlike IFT20, GMAP210 is not absolutely required for ciliary assembly. Shorter cilia, with two-third length of normal cilia, were still assembled upon the loss of GMAP210 (Follit et al., 2008). Complete loss of IFT20 blocked ciliary assembly precluding the analysis of vesicle trafficking. Partial loss of IFT20 did not prevent cilia assembly; however, it impaired the cilium-targeted vesicular trafficking of polycystin-2, a ciliary transmembrane protein. In GMAP210 mutant cells, ciliary levels of polycystin-2 also decreased, which indicated the interplay of IFT20 and GMAP210 in regulating the cilium-targeted vesicular transport from the Golgi. Both phenotypes, upon loss of GMAP210, might be explained by the fact that the members of the golgin family function in a redundant manner, co-operating on the surface of the Golgi to tether vesicles or Golgi membranes, and loss of a single golgin might be compensated for other golgins with similar tethering specificity.

Indeed, GMAP210 acts in a partially redundant manner with GM130, another *cis* face resident golgin, to ensure efficient anterograde cargo delivery to the *cis-*Golgi (Roboti et al., 2015). GM130 could form a new complex with VPS15, whose missense mutation was identified in a family with a ciliopathy and led to shorter cilia. The number of IFT20-associated vesicles derived from the Golgi decreased in VPS15-mutant cells. Moreover, the interaction of GM130 with VPS15 and GMAP210 with IFT20 occurred both in the control and VPS15 missense mutant cells; however, the interaction of GM130 with IFT20 was only detected in the control, but not in the mutant cells. Therefore, VPS15-GM130 might serve as a platform to release IFT20-positive vesicles (Stoetzel et al., 2016). IFT20 might interact with these two functionally redundant golgins, GMAP210 and GM130, sequentially or simultaneously to promote the formation and release of IFT20-positive vesicles (Figure 1A).

In addition to GMAP210 and GM130, other golgins might also interact with IFT20 to facilitate vesicular trafficking in specific cell types. In photoreceptor cells, IFT20 could also be found on the TGN and post-Golgi vesicles that transport ciliary membrane proteins, such as opsin, into the outer segment that is a modified cilium (Keady et al., 2011). Deletion of IFT20 caused opsin accumulation at the Golgi; however, deletion of IFT140, a subunit of IFT-A complex, caused opsin accumulation in the plasma membrane of the inner segment (Crouse et al., 2014). These data suggested that other IFT subunits were also involved in vesicular trafficking targeted to the cilium (Sedmak and Wolfrum, 2010). Moreover, TGN is an active site to deliver cilium-targeted, as well as plasma membrane-targeted and endosome-targeted vesicles. Some components of these three vesicular trafficking pathways are shared. For example, FAPP2 is a resident TGN protein whose abolishment resulted in impaired ciliogenesis and accumulation of vesicles between the apical membrane and the centrioles (Vieira et al., 2006). Additionally, AP-1, identified initially as a clathrin-associated adapter, also participated in the transport of several ciliary membrane proteins from TGN to Rab8-positive vesicles (Kaplan et al., 2010). It would be interesting to determine whether IFT20 is also involved in plasma membrane-targeted and endosome-targeted vesicular trafficking pathways, functioning as a shared protein in Golgi-derived vesicular trafficking in some specific cell types.

#### IFT20 and the Centrosome

On completion of the early events of ciliogenesis, such as establishment of DAPs, formation of preciliary membrane compartment, recruitment of IFT components, and assembly of the TZ, the ciliary axoneme starts elongating. Progress of these early events is mostly dependent on the interaction of various vesicles with the mother centriole. Moreover, the centrosome-directed vesicular trafficking of signaling proteins to a membrane patch presumably inaugurated a ciliary precursor, facilitating more efficient signal transduction (Sung and Leroux, 2013).

The establishment of mammalian DAPs of the mother centriole is initiated by the recruitment of C2CD3, followed by other components, such as CCDC41 and Cep164, thus forming a 9-fold symmetrical radial finger-like protrusion with a diameter of over 500 nm, which provides a broad platform to facilitate the vesicle-centriole association (Bowler et al., 2019). Among these components of DAPs, CCDC41, comprising multiple coiled-coil domains, has been proven to anchor the IFT20-positive vesicles to the mother centriole. Knockdown of CCDC41 remarkably inhibited the recruitment of IFT20 to the centrosome and prevented ciliogenesis at the ciliary vesicle docking step (Joo et al., 2013), thereby suggesting that the interaction of IFT20-associated vesicles with DAPs is essential for ciliogenesis (Figure 1A). The colocalization of IFT52 with transitional fibers at the basal body suggested that the transitional fibers derived from DAPs also act as the docking site for other IFT subunits (Deane et al., 2001).

Cilia-targeted vesicles derived from the recycling endosomes or the Golgi must be transported in a specific order to ensure the precise assembly of cilia (Nachury et al., 2007; Knodler et al., 2010; Westlake et al., 2011). Using time-lapse and transmission electron microscopy, Westlake and his colleagues proved that the accumulation of IFT20-positive vesicles at DAPs followed the EHD1/EHD3-dependent assembly of small distal appendage vesicles (DAVs) and occurred before Rab8-dependent post- ciliary vesicles (CV) extension. With the loss of EHD1/EHD3, IFT20-positive vesicles failed to accumulate at the mother centriole, and ciliogenesis was disrupted; however, such accumulation was independent of Rab8-positive vesicular trafficking (Lu et al., 2015). Another study performed in the Malicki’s laboratory revealed that IFT54, a subunit of IFT-B2 complex with an N-terminal calponin homology (CH) domain and a C-terminal coiled-coil domain, directly interacted with IFT20 and linked IFT20 to Rab8 via Rabaptin5. The complex of IFT20–IFT54–Rabaptin5–Rab8 may provide a bridge between the IFT subunits and Rab8-associated vesicles (Figure 1A; Omori et al., 2008). Thus, the IFT20-positive vesicles reached the mother centriole earlier than those associated with Rab8, and IFT54 mediated the interaction between them. Moreover, IFT54 seemed to be responsible for the incorporation of IFT20-associated vesicles into IFT trains at the basal body (Zhu et al., 2017).

The noteworthy research from the Pazour’s laboratory revealed that the mother centriole pool of IFT20 is partially responsible for the basal body localization of pallidin, a subunit of BLOC-1 complex (the biogenesis of lysosome-related organelles complex-1) that functions in endosome sorting, indicating that IFT20 might mediate the anchoring of endosomal BLOC-1-dependent vesicles at the basal body (Figure 1A; Monis et al., 2017). Such interaction appeared to be involved in sperm development. *Ift20^–/–^* knockout mice are infertile, with significantly reduced sperm count and motility. The results of electron microscopy revealed an increase in the cytoplasmic vesicles and a decrease in the lysosomes (Zhang et al., 2016). Lysosome maturation was associated with the BLOC-1 complex (Cai et al., 2010; Yuzaki, 2010), indicating the relevance of IFT20 and the BLOC-1 complex. Moreover, in IFT20-deficient cells, a defect was observed during autophagic clearance, and lipid droplets accumulated mainly due to the dysfunction of the lysosome (Zhang et al., 2016; Finetti et al., 2020).

Mechanistically, IFT20 was shown to regulate the retrograde traffic of the cation-independent mannose-6-phosphate receptors (CI-MPR) to the TGN by coupling the recycling CI-MPRs to the microtubule motor dynein. These receptors are essential for the lysosomal targeting of acid hydrolases, which are required for the degradation function of the lysosome (Figure 1C; Finetti et al., 2020). IFT20 also interacted with Exo70 and Sec8, two components of the exocyst complex tethering vesicles at target sites before membrane fusion (Heider and Munson, 2012; Heider et al., 2016); however, the localization of Exo70 and Sec8 at the basal body was independent of IFT20. The association of IFT20 and components of the exocyst complex might bridge some membrane proteins independent of IFT-associated vesicles to IFT trains at the basal body (Figure 1A).

The assembly and disassembly of cilia are coordinated with the cell cycle. The cilium disassembles at the onset of mitosis, releasing centrioles to function as the microtubule organizing center (MTOC) at the poles of the spindle apparatus (Kim and Tsiokas, 2011). When cells entered mitosis, the Golgi was dispersed into small structures that coalesced with the centrosomes, and part of IFT20 remained associated with the centrosomes (Follit et al., 2006). Moreover, deletion of IFT20 specifically in kidney collecting duct cells (germline deletion of IFT20 caused embryonic lethality) not only disrupted the cilia formation but also caused rapid postnatal cystic expansion of the kidneys due to misorientation of the mitotic spindle (Jonassen et al., 2008). During cytokinesis, the ingression of the cleavage furrow constricts the cytoplasm, and the spindle microtubules are transformed as the intercellular bridge connecting the two daughter cells. In the middle of the intercellular bridge, the midbody, a temporary structure with overlapping antiparallel microtubule bundles, is formed. Interestingly, IFT20, IFT88, and Rab8 were found to localize at the midbody during cytokinesis (Bernabe-Rubio et al., 2016). After cytokinesis, the midbody was inherited by one of the daughter cells as a remnant. When the remnant, carrying Rab8, IFT, and exocyst components, moved closer to the centrosome, a primary cilium began to assemble; however, whether the materials from the remnant contributed to form ciliary vesicles during early ciliogenesis has not yet been determined (Paridaen et al., 2013; Bernabe-Rubio et al., 2016).

#### IFT20 and Ciliary Membrane Proteins

It was reported that membrane proteins targeted to the cilium depended on ciliary targeting sequences (CTSs) within their cytosolic domain. Based on the truncation localization assays, several proposed CTSs, including the RVxP motif (polycystin-2), VxPx motif (rhodopsin), and the AxS/AxQ motif (somatostatin receptor 3, serotonin receptor 6, and melanocortin-concentrating hormone receptor 1), were identified (Pazour and Bloodgood, 2008; Nachury et al., 2010); however, unlike the nuclear localizing sequence (NLS), a consensus sequence in the ciliary receptors was not found.

Two well-known ciliary receptors interact with IFT20, polycystin-2 (associated with the human autosomal dominant polycystic kidney disease), and opsins; however, a weak association was observed between IFT20 and CTS in these two receptors. IFT20 could interact robustly with the cytoplasmic tail of opsin, but the deletion of the last four residues, which contained a VxPx motif, did not affect the binding of IFT20, suggesting that other motifs in the cytoplasmic tail were responsible for the identification by IFT20. Moreover, Arf4, a small GTPase, directly bound the VxPx motif and regulated the opsin association with the TGN (Deretic, 2006; Mazelova et al., 2009). These results suggested that there might be interconnected crossing pathways for transporting membrane proteins to the cilium, IFT20-dependent vesicular trafficking being one of these.

In the ciliated neurons of *C. elegans*, IFT20 neither localizes to the Golgi apparatus nor physically interacts with SQL-1, the homolog of GMAP210 (Broekhuis et al., 2013). Additionally, not all transmembrane proteins targeting cilia require IFT proteins. For example, ODR-10 (odorant receptor) in *C. elegans* requires AP-1, clathrin, and Rab8 to be transported into AWB cilia (a specific type of amphid channel cilia with elaborated wing or fork morphology in *C. elegans* sensory neurons), but not IFT proteins (Kaplan et al., 2010). Thus, the role of IFT20 in trafficking ciliary membrane proteins might not be conserved in *C. elegans*.

### IFT172 and Vesicular Trafficking

Vesicle formation requires membrane-deforming proteins and complexes (Field et al., 2011). Recently, the *in vitro* study of IFT172 demonstrated that a direct interaction exists between the IFT particles and membranes. IFT172 is the largest IFT subunit and is essential for ciliogenesis, which belongs to the peripheral IFT-B2 complex. It has seven WD domains for β-propeller and TRP domains for α-solenoid, shares a similar domain organization with COP, and demonstrates its potential as a membrane-deforming protein.

The initial functional characterization of IFT172 was carried out on a temperature-sensitive mutant *fla11*^*ts*^, in which *fla11* encodes IFT172 with a single amino acid mutation from a conserved leucine to proline. When this mutant was shifted to the restrictive temperature, IFT-B particles accumulated at the ciliary tip, indicating a defect in IFT-train remodeling. Therefore, IFT172 might mediate the re-assembly of IFT-B with the IFT-A complex at the ciliary tip presumably via the interaction with EB1, a microtubule plus end binding protein (Pedersen et al., 2005; Sloboda, 2005). In humans, IFT172 mutations were identified in a consanguineous family with the typical Bardet–Biedl syndrome (Schaefer et al., 2016), a type of ciliopathy in which the mutated proteins are mostly involved in membrane trafficking.

Recent studies reported that IFT172 could interact with membranes and was able to remodel large membranes into small vesicles, thus suggesting that the interaction of IFT172 and membrane lipids could provide a partial thermodynamic force to achieve fission *in vivo*. IFT172 might recognize the membrane surface through its charged N-terminal β-propeller blade surface. This domain also acts as the binding site of IFT57, another subunit of the IFT-B2 complex. Less IFT172 interacts with membrane surfaces when IFT57 is added in the liposome co-sedimentation assay, indicating a mutually exclusive relationship between IFT57 and membrane structures; however, it remains unclear when the IFT trains arrive at the tip, whether another interaction with EB1 might facilitate the release of IFT172 from IFT trains and the rebinding of the ciliary tip membrane. As secretory events occur at the ciliary tip via budding and IFT172 can bend membranes, it is also unclear whether the parking of IFT172 at the tip is involved in these secretory events through changing the local curvature of the ciliary tip membrane. Immunofluorescence localization results revealed that large foci of IFT172 could be observed around the mother centriole at the initiation stage of ciliogenesis, implying that IFT172-lipid association may be present in the entire cilium structure (Wang et al., 2018).

### IFT22, IFT25, and IFT27 and Vesicular Trafficking

The Rab GTPases are central components of the trafficking machinery, define the identity of intracellular vesicles, and control the direction of both inward and outward flow of cargoes (Hutagalung and Novick, 2011). The discovery that three IFT proteins (IFT22, IFT25, and IFT27) are Rab-like GTPases highlights the vesicular trafficking-related function of IFT subunits (Schafer et al., 2006; Qin et al., 2007; Adhiambo et al., 2009; Keady et al., 2012; Liew et al., 2014; Dong et al., 2017; Wachter et al., 2019).

IFT22, identified initially as Rab-like 5 protein (RabL5), is required for cilium formation in *Trypanosoma* (Adhiambo et al., 2009; Wachter et al., 2019), but not in *C. elegans* where it modulates insulin signaling (Schafer et al., 2006). Additionally, a recent study in *Trypanosoma* revealed that the association of IFT22 with IFT74/81 was essential for cilium construction, but IFT22 GTP loading was not strictly required (Wachter et al., 2019).

IFT25 (RABL2) is another Rab-like GTPase in the IFT-B1 complex, forms a heterodimer with IFT27, and is essential to maintain IFT27 stability *in vivo*. Unlike other core IFT-B1 subunits, IFT25 is dispensable for ciliogenesis in most organisms. IFT25 also regulates the transport of Hedgehog signaling proteins in vertebrate cilia (Keady et al., 2012). In humans, two highly similar paralogs, RABL2A and RABL2B, have been identified (Martin et al., 2002), of which one is recruited to DAPs by a mother centriole protein CEP19. The GTP-loading RABL2B at the basal body could bind to the IFT train to initiate IFT (Kanie et al., 2017). The vesicular trafficking function of IFT25 might depend on IFT27 to form a docking site for the BBSome on the retrograde IFT trains, as the BBSome and associated cargoes accumulated in IFT25 and IFT27 mutant *Chlamydomonas* and mammalian cells (Eguether et al., 2014; Liew et al., 2014; Dong et al., 2017).

Differing from the spindle pole localization of IFT52 (Deane et al., 2001), IFT27 was found to localize at the cleavage furrow in *Chlamydomonas* during mitosis (Wood et al., 2012). Vesicular localization of IFT27 surrounding the cleavage furrow was confirmed by immunogold labeling and transmission electron microscopy. This localization pattern might be attributed to the vesicular transport function of IFT27, as the maturation of the cleavage furrow was associated with membrane supplementation. IFT27 returned to the basal bodies when the furrow matured and cells underwent cleavage. A similar localization pattern of other IFT subunits at the midbody was also recorded in mammalian cells (Bernabe-Rubio et al., 2016). Moreover, the crystal structure of IFT27 resembles those of Rab8 and Rab11 (Bhogaraju et al., 2011). Therefore, after Rab8 or Rab11 vesicles reached the basal body, IFT25/27 might replace them to bind the vesicles (Figure 1A).

Rab GTPases regulate nearly all steps of membrane trafficking from the formation of transport vesicles at the donor membrane to their fusion with the target membrane (Hutagalung and Novick, 2011); however, the regulatory function of Rab-like GTPases of several IFT subunits seems to be poorly understood, presumably because they are not typical GTPases due to the lack of a membrane-targeting prenylation site. However, these Rab-like GTPases of IFT subunits could actively interact with the BBSome complex, whose mutation causes the Bardet–Biedl syndrome (Blacque and Leroux, 2006). The BBSome core complex was initially discovered in mammalian cells (Nachury et al., 2007). Mutations in BBSome components have less effect on the primary cilium assembly but fail to promote ciliary membrane protein trafficking via interaction with Rab8 and Rab11 vesicles (Mykytyn et al., 2004; Berbari et al., 2008; Jin et al., 2010). In *Chlamydomonas*, the BBSome functions as cargo adaptors of IFT (Lechtreck et al., 2009); however, in *C. elegans*, the BBSome is required for IFT assembly and normal ciliogenesis (Ou et al., 2005; Wei et al., 2012). Similar to IFT subunits, the components of the BBSome also share structural similarities with COPI, COPII, and clathrin coats (Jin et al., 2010), suggesting that the BBSome proteins are likely to have co-evolved with IFT proteins to augment the versatility and specificity of the ciliary cargoes.

Not all IFT trains are loaded with the BBSome complex, and the functions of the BBSome in ciliary assembly are distinct in several models. For example, BBS4 and BBS5 are two components of the BBSome. In *Chlamydomonas*, BBS4 mutants show normal flagella, but knockdown of BBS5 leads to the absence of flagella (Li et al., 2004; Lechtreck et al., 2009). In mammalian cells, BBS4 through its TRP repeats acts as a bridging factor between PCM-1 and dynein to bring proteins to the centrosome (Kim et al., 2004), whereas BBS5 binds to phosphoinositides through its two pleckstrin homology-like domains (PH-like) and is critical for ciliogenesis (Nachury et al., 2007). In zebrafish, knockdown of BBS4 and BBS5 result in similar phenotypes, including disruption of Kupffer’s vesicle, predisposition to organ laterality, and delayed intracellular retrograde transport (Yen et al., 2006). In *C. elegans*, these two proteins show unexpected functional redundancy in regulating the ciliary removal of various sensory receptors, and co-depletion of BBS-4 and BBS-5 disrupts the lysosome-targeted degradative sorting of ciliary sensory receptors in *C. elegans* (Xu et al., 2015). Therefore, although the BBSome subunits are conserved through ciliated organisms, the alternative mechanisms might exist in different organisms, and more elaborate function of the BBSome complex in different organisms does still need more exploration.

## The Vesicular Trafficking Function of IFT Subunits in Non-Ciliated Cells

As certain human diseases are associated with cilia defects, immense research is conducted in this field; however, notably, several ciliary proteins, including IFT proteins, are found at other sites outside the cilium and are speculated to possess extraciliary functions. It is difficult to determine that an observed phenotype owes to defective cilia or/and some defective extraciliary function (Baldari and Rosenbaum, 2010; Yuan and Sun, 2013; Vertii et al., 2015; Hua and Ferland, 2018a, b). Extraciliary functions of the ciliary proteins have been reported to be involved in various aspects of cell activities, such as cell cycle regulation (Qin et al., 2007), cytoskeletal and migration regulation (Nishita et al., 2017), establishment of polarity (Toriyama et al., 2016), cellular metabolism (Lee et al., 2018), secretion of extracellular matrix (Noda et al., 2016), and the regulation of transcription factors to be translocated into nucleus (Vuong et al., 2018). Therefore, to better understand the pathogenesis of ciliopathies, it is necessary to elucidate the additional extraciliary roles of these ciliary proteins. Herein, we reviewed our present understanding of the extraciliary functions of IFT subunits with a focus on vesicular trafficking.

### IFT Subunits and Immune Synapse Formation in T Cells

It is challenging to study the extraciliary role of IFT subunits independent of the cilium, as the cilium exists in most eukaryotic cells; however, hematopoietic cells, such as lymphoid and myeloid cells, are one of the few cell types that lack a cilium but express IFT proteins. Quantitative real-time (RT)-PCR analysis revealed that all components of the IFT system are expressed in T cells (Finetti et al., 2014).

When the native T-cell encounters an antigen-presenting cell (APC) carrying cognate peptide ligand, the MTOC derived from the centrosome will be *trans-*located beneath the T-cell and APC contact area, which will mature into the immune synapse (IS) (Figure 1C). The Golgi and other vesicular compartments also orient toward the IS, which facilitates the targeted delivery of signaling molecules from endosomes or TGN to the IS (Sancho et al., 2002; Martin-Cofreces et al., 2008). The T-cell receptors (TCR) are recruited to the IS by two pathways. One is by lateral mobility from the plasma membrane-associated pools within seconds of T-cell activation, and the other is by polarized trafficking to the IS via the recycling endosome pathway within few minutes of T-cell activation (Bonello et al., 2004; Cemerski and Shaw, 2006; Finetti and Baldari, 2013).

The discovery that IFT proteins played an intracellular membrane trafficking role in the non-ciliated cells originated from the immunofluorescent analysis of IFT20 in T cells. Most of IFT20 localized at the *cis-*Golgi and the centrosome; however, a limited amount of IFT20 was also observed at the TGN, as well as at early endosomes (marked by Rab5) and recycling endosomes (marked by Rab4 or Rab11), strongly suggesting that IFT20 is dynamically transported among these endomembrane structures. After the activation of T cells, IFT20 was found to cluster at the IS, concomitantly with the reorientation of the Golgi and centrosome, indicating that IFT20 might participate in the polarized vesicular trafficking toward the IS during T-cell activation. TCR/CD3 and TfR (transferrin receptor), not CXCR4, failed to cluster at the IS when IFT20 was knocked down. Therefore, IFT20 was explicitly required for recycling of the specific receptors to the IS, with the assistance of IFT52, IFT57, and IFT88 (Figure 1C; Finetti et al., 2009, 2014). IFT57, the first reported IFT subunit interacting with IFT20 using the two-yeast hybrid assay (Baker et al., 2003), also revealed a vesicular localization around the centrosome and was clustered to the IS during activation, but did not colocalize with the Golgi, suggesting that IFT57 might be recruited to the IFT20-tagged vesicles in order to assist vesicular trafficking. Some of the critical components required for polarized TCR recycling may be pre-assembled on the endosome surface that contains TCR cargoes; however, it was not clear which proteins mediated the interaction of TCR-containing endosomes with IFT subunit-tagged vesicles. Based on the research of ciliogenesis and endomembrane trafficking, Rab GTPases seemed to be good candidates (Markgraf et al., 2007; Sung and Leroux, 2013).

The recycling of TCR is required for sustained signaling at the IS. Following internalization into early endosomes marked by Rab5, TCR-containing vesicles are rapidly redirected to the cell surfaces in Rab4 marked recycling endosomes. Alternatively, they are targeted to the pericentrosomal recycling compartment, identified by Rab11, and are then transported to the cell surfaces using a longer route (Grant and Donaldson, 2009). IFT20 was proven to promote the transit of internalized TCRs from early to recycling endosomes (Figure 1C), indicating an interplay between IFT20 and Rab-based regulatory machinery in the polarized trafficking of TCR.

In addition to Rab proteins and three IFT subunits (IFT52, IFT57, and IFT88), some new players were identified in IFT20 interactomes, which included IFT54, GMAP210, subunit-3 of Arp2/3 complex (ARPC3), subunit-1 of COP9 signalosome (CSN1), and ERGIC-53. Moreover, loss of IFT54, ARPC3, or ERGIC-53 led to failure of endosomal TCR and TfR accumulation at the IS, which was in accordance with that observed in IFT20-deficient T cells; this greatly increased the complexity and diversity of the vesicular trafficking pathways where IFT20 participated (Galgano et al., 2017). Another axis of IFT20–IFT54–microtubule was found to be exploited to move the recycling endosomes to the IS in T cells (Bizet et al., 2015). For the interactor GMAP210, a recently published article showed that GMAP210 localized intracellular vesicular pools, as well as the Golgi, and could convey specific vesicles containing linker for activation of T cells (LAT) to the IS. More interestingly, in a model of ectopic expression of LAT in ciliated cells, GMAP210 tethering activity controlled the delivery of LAT to the cilium, which highlights the similarities and intersection of vesicular transport in both ciliated and non-ciliated cells (Zucchetti et al., 2019).

Importantly, both the *in vivo* and *in vitro* results from two independent laboratories confirmed the function of IFT20 in T cells. In mice with CD4 T-cell specific knockout (KO) of IFT20, LAT failed to be recruited into the IS (Vivar et al., 2016). Another report revealed that IFT20 was crucial for the early, but not for later development of T cells. When IFT20 was specifically knocked out in the early and later stages of T-cell development by crossing *IFT20*^*flox/flox*^ mice with Lck-Cre (representative gene of early stage) or CD4-Cre (representative gene of later stage) transgenic mice, no differences in the body size and morphology of immune organs were observed in these two knockout strains; however, the number of CD4- and CD8-positive cells was significantly decreased only in *Lck-Cre/IFT20^*flox/flox*^*, which also demonstrated the downregulation of some crucial cytokines (IL-1β, IL-6, and TGF-β1) and abnormal immune behaviors, such as less severity of collagen-induced arthritis (CIA) symptoms and weaker inflammation in the paws, indicating that normal differentiation of T cells was disrupted upon loss of IFT20 (Yuan et al., 2014). Collectively, these findings in T cells enhanced our knowledge of the intracellular vesicular trafficking function of IFT subunits beyond the ciliogenesis.

### IFT Subunits and Synaptic Vesicular Trafficking in Non-ciliated Neuron

The evidence that IFT subunits are required for recycling TCR and LAT to the IS tempted us to hypothesize that IFT subunits also participated in the development of neurite, a sensing, secreting, and polarized structure.

The observations by immunoelectron microscopy in retinas revealed that IFT20, IFT52, IFT57, IFT88, and IFT140 not only localized at the connecting cilium but also localized at defined periciliary membranes in photoreceptors. Unexpectedly, a non-classic IFT system comprising IFT20, IFT52, and IFT57 also participated in the vesicular transport targeted to the postsynaptic dendritic terminal in secondary retinal neurons, which lacked cilia (Figure 1B) (Sedmak and Wolfrum, 2010). Thereafter, the same three proteins were once again found during targeted vesicular trafficking to IS. Both of these findings suggested that polarized protrusion structures, such as cilia, growth cones, dendritic spines, ISs, and migration podosomes, might share a similar building design in polarized vesicular trafficking (Finetti and Baldari, 2013; Hua and Ferland, 2018b).

### IFT Subunits and Cancer Cells

In the past decade, several studies have demonstrated that cilia also played essential roles in tumorigenesis (Han et al., 2009; Wong et al., 2009). Loss of primary cilia had been detected in an early stage of some cancers, such as breast, pancreatic, and renal cell carcinoma (Schraml et al., 2009; Seeley et al., 2009; Yuan et al., 2010; Kim et al., 2011; Basten et al., 2013; Hassounah et al., 2013). Therefore, cancer cells without a cilium might be appropriate models for investigating the extraciliary functions of IFT subunits.

Oncogenesis is accompanied by overactivation or inactivation of various signaling pathways, among which Ror2-Wnt5a presents overactivation (Endo et al., 2015). Recently, a study in human osteosarcoma cell lines (SaOS2) lacking cilia revealed that IFT20 is a new component for Ror-Wnt5a signaling and that it regulates the nucleation of Golgi-derived microtubules via interaction with the GM130-AKAP450 complex. The vertebrate Golgi complex comprises stacked cisternae that are laterally linked to form the Golgi ribbon; knockdown of IFT20 disrupts the ribbon structure of the Golgi and impairs the invasiveness of osteosarcoma cells (Nishita et al., 2017). These data suggested that in non-ciliated tumor cells, IFT20 may be involved in tumor progression. IFT88, another subunit of IFT-B complex, was also reported to influence cell migration via regulating microtubule dynamics at the leading edge of migrating cells, which is independent of cilia (Boehlke et al., 2015). In contrast, the phenotype of IFT88 in thyroid cancer was more relevant to the mitochondrial oxidative function. Gene expression patterns in IFT88-deficient thyroid cancer cells favored glycolysis and lipid biosynthesis (Lee et al., 2018), which was beyond our understanding of the functions of IFT subunits and prompted us to re-examine the molecular mechanism of ciliopathies.

### IFT Subunits and Cellular Secretion

Mutation of three subunits of the IFT-A complex (IFT122, IFT140, and IFT144) resulted in human pleiotropic ciliopathies, Sensenbrenner and Jeune syndromes, in which the pathological features included skeletal development abnormalities (Walczak-Sztulpa et al., 2010; Ashe et al., 2012; Miller et al., 2013). This observation highlights the critical role of IFT subunits in skeletal development, but the underlying mechanism remains unclear.

While studying mice with neural crest-specific deletion of IFT20, the Komatsu and his colleagues unexpectedly found that in addition to the failure of ciliogenesis, the intracellular collagen transport was also disrupted, thus leading to osteopenia in the facial region (Noda et al., 2016). The deficiency of IFT20 in cranial neural crest (CNC)-derived cells severely attenuated the process of mineralization, due to delayed transport and secretion of type I collagen.

Because the mice with neural crest-specific deletion of IFT20 died shortly after birth due to difficulties in feeding and breathing, another mouse line with chondrocyte-specific deletion of IFT20 at juvenile-to-adult stages was obtained (Kitami et al., 2018). In accordance with the previous studies, the maturation process of condylar cartilage was disrupted, owing to the lower amount of collagen type X and reduced proliferation. In normal chondrocytes, the Golgi will be expanded during the secretion of the cartilaginous matrix; however, the Golgi size decreased in IFT20-KO chondrocytes, indicating that the reduced amount of cartilaginous matrix in condylar cartilage partially contributed to the abnormal Golgi size upon loss of IFT20.

Interestingly, a similar defect also appeared in GMAP-210 KO mice in which Golgi vesiculation and impaired cargo secretion occurred; however, this phenotype was only evident in specific cell types, such as chondrocytes that were responsible for cartilage and bone deposition. Mutations in human GMAP210 also caused neonatal lethal skeletal dysplasia achondrogenesis type 1A; whether this arose from reduced secretion of extracellular matrix proteins remains to be ascertained (Smits et al., 2010; Roboti et al., 2015). Collectively, the physiological interaction of IFT20-GMAP210 functions not only in ciliogenesis but also in bone development with the converging theme of intracellular vesicular trafficking.

Whether additional secretory cargoes need IFT20-associated vesicular transport remains unclear. Our previous studies in BLOC-1 complex demonstrated that loss of Bloc1s1, a subunit of BLOC-1 complex, impaired the secretion of surfactant in the swim-bladder of zebrafish (Chen et al., 2018) and a novel interaction of IFT20 and BLOC-1 complex was identified in Pazour’s laboratory (Monis et al., 2017). Therefore, detecting whether the surfactant was included in IFT20 cargoes might provide a new perspective in understanding the intersections between different vesicular transport pathways.

## Conclusion and Future Directions

Initially, IFT was regarded as an exclusive transport system essential for ciliogenesis (Kozminski et al., 1993). Recent research and bioinformatic data highlighted that the IFT system belonged to the family of the COP complex, which is the essential component of intracellular vesicular trafficking (Jekely and Arendt, 2006). The transport function of IFT in ciliogenesis may have evolved from the general intracellular vesicular trafficking function; however, two caveats were present: (1) none of the IFT subunits possess transmembrane domains, lipid modifications, or lipid-binding domains; (2) the cilium is not entirely a membrane-bound organelle. Both caveats hindered the connection between IFT subunits and vesicular trafficking. To date, this connection has gained immense attention and more evidence has been obtained to explore this association. The most direct evidence was IFT20, localizing at the Golgi, the center of the endomembrane system. In addition, IFT20 can use a canonical intracellular vesicular trafficking pathway, such as the recycling endosome pathway, to transport specific receptors to the ciliary membrane in conjunction with tethering factors, Rab GTPases, and exocyst subunits. This strongly confirmed the association of IFT subunits with vesicular trafficking and indicated that IFT may be an extension of the vesicular trafficking pathway.

Besides the subunits of the IFT-B complex, there is increasing evidence that IFT-A subunits are required for the transport of specific ciliary membrane proteins into or outside the cilium. A recently published article revealed that when truncated IFT140 lacking WD40 repeats was expressed in the null mutant, the axonemes of these cilia had a normal ultrastructure, but the composition of membrane and matrix were abnormal with a decrease in small GTPases, lipid-anchored proteins, and cell signaling proteins (Picariello et al., 2019), indicating that IFT-A subunits might also be specialized for importing the membrane-associated proteins.

As a cilium is found in nearly all types of human cells, notably, the ciliary defects could result in numerous human diseases, including polycystic kidney diseases, skeletal abnormalities, blindness, obesity, and cancer, some of which were attributed to the mutation of IFT components; however, the cilium may not be the sole mediator of these defects, and certain phenotypes may be caused by the dysfunction of IFT particles at the extraciliary sites. Therefore, the intracellular vesicular trafficking function of IFT subunits we reviewed is congruous with an emerging concept; in addition to their well-established roles in ciliary assembly, IFT subunits may have more general roles in vesicular trafficking.

Nevertheless, the involvement of IFT subunits in vesicular trafficking has not been fully elucidated. The structural analysis of IFT particles and identification of the transiently interacting proteins of IFT subunits might help understand the IFT–vesicle interactions at the molecular level. As only a small fraction of IFT subunits is associated with vesicular trafficking, and as this interaction is quite dynamic, super-resolution and electron microscopy are required to study this process. Undoubtedly, the aforementioned research will serve to illuminate the interconnected vesicular trafficking pathways in regulating cellular homeostasis.

## Author Contributions

HY wrote the first draft of the manuscript. KH revised the manuscript.

## Conflict of Interest

The authors declare that the research was conducted in the absence of any commercial or financial relationships that could be construed as a potential conflict of interest.
